# Dimethyl fumarate modulates the regulatory T cell response in the mesenteric lymph nodes of mice with experimental autoimmune encephalomyelitis

**DOI:** 10.3389/fimmu.2024.1391949

**Published:** 2024-05-03

**Authors:** Amanda D. R. Lima, Breno B. Ferrari, Fernando Pradella, Rodrigo M. Carvalho, Sandra L. S. Rivero, Raphael P. S. Quintiliano, Matheus A. Souza, Natália S. Brunetti, Ana M. Marques, Irene P. Santos, Alessandro S. Farias, Elaine C. Oliveira, Leonilda M. B. Santos

**Affiliations:** ^1^ Unidade de Neuroimunologia, Dept.Genética, Evolução, Microbiologia e Imunologia, Universidade Estadual de Campinas (UNICAMP), Campinas, Brazil; ^2^ Departamento de Citometria do Centro de Hematologia e Hemoterapia da UNICAMP, Universidade Estadual de Campinas (UNICAMP), Campinas, Brazil; ^3^ Technology Faculty of Sorocaba- Paula Souza State Center of Technological Education, Sorocaba, Brazil; ^4^ Brazilian National Institute of Science and Technology on Neuroimmunomodulation, (INCT-NIM), National Council for Scientific and Technological Development (CNPq), Brasilia, Brazil

**Keywords:** dimethyl fumarate, experimental autoimmune encephalomyelitis, type 1 regulatory T cells, anti-inflammatory cytokines, gut draining lymph nodes

## Abstract

Dimethyl fumarate (DMF, Tecfidera) is an oral drug utilized to treat relapsing-remitting multiple sclerosis (MS). DMF treatment reduces disease activity in MS. Gastrointestinal discomfort is a common adverse effect of the treatment with DMF. This study aimed to investigate the effect of DMF administration in the gut draining lymph nodes cells of C57BL6/J female mice with experimental autoimmune encephalomyelitis (EAE), an animal model of MS. We have demonstrated that the treatment with DMF (7.5 mg/kg) significantly reduces the severity of EAE. This reduction of the severity is accompanied by the increase of both proinflammatory and anti-inflammatory mechanisms at the beginning of the treatment. As the treatment progressed, we observed an increasing number of regulatory Foxp3 negative CD4 T cells (Tr1), and anti-inflammatory cytokines such as IL-27, as well as the reduction of PGE2 level in the mesenteric lymph nodes of mice with EAE. We provide evidence that DMF induces a gradual anti-inflammatory response in the gut draining lymph nodes, which might contribute to the reduction of both intestinal discomfort and the inflammatory response of EAE. These findings indicate that the gut is the first microenvironment of action of DMF, which may contribute to its effects of reducing disease severity in MS patients.

## 1 Introduction

Dimethyl fumarate (DMF, TECFIDERA, Biogen - USA) was approved as a first-line oral therapy for relapsing-remitting multiple sclerosis (MS). Its efficacy arises from neuroprotective and immunomodulatory effects, alongside a favorable benefit-risk profile. DMF’s neuroprotective benefits are primarily attributed to the activation of the Nrf2 antioxidant pathway in central nervous system (CNS) cells (1) and glutathione recycling (2). Additionally, its immunomodulatory properties are associated with the activation of the HCAR2/GPR109 pathway in microglia (3, 4) and the inhibition of the pro-inflammatory transcription factor NF-kB (5, 6).

Lymphopenia, flushing, and gastrointestinal events are the most common adverse effects of the treatment with DMF. DMF is converted into its main metabolite, monomethyl fumarate (MMF), by esterases from intestinal epithelial cells (7). The intestinal discomfort observed by oral administration of DMF seems to be the result of an inflammatory response induced by the treatment. These symptoms appear at the beginning and are reduced during treatment (8, 9), suggesting that an anti-inflammatory response is also activated by the DMF.

An important property of the cells of the intestinal mucosa is to prevent an exaggerated immune response, such as allergies or inflammation, to orally acquired antigens. The mesenteric lymph nodes are constantly draining immune cells from the gastrointestinal tract, either in a steady state or under inflammatory conditions, resulting in the induction of tolerance or immune cell activation. In the experimental autoimmune encephalomyelitis (EAE), the animal model for MS, the induction of regulatory T cells was demonstrated in classical experiments of adoptive cells transfer. The transfer of mesenteric lymph node cells and Peyer’s patch cells from rodents tolerized to myelin basic protein (MBP) protected against actively induced EAE (10–12). Here, we provide evidence that treatment with DMF gradually activated the expression of both regulatory T cells and IL-27 production in the mesenteric lymph nodes of mice with EAE.

## 2 Methods

### 2.1 Animals

Wild-type C57BL/6J female mice, 8 weeks old, were used in the experiments. They were obtained at the Multidisciplinary Center for Biological Research in the Area of Science in Laboratory Animals (CEMIB – UNICAMP), and they were housed in a specific pathogen-free facility of the Institute of Biology (UNICAMP). The temperature was kept between 21-23°C, and a standard light-dark cycle of 12 h was applied. The animals received sterile drinking water and food (Nuvilab - Quimtia) ad libitum. No animal was excluded during experiments. Mice were littermates and were randomly allocated into cohorts. Mice health was monitored daily. The study followed the updated recommendations of the National Council for the Control of Animal Experimentation (CONCEA), and it was approved by the UNICAMP Animal Ethics Committee (CEUA/UNICAMP), with registration number 4884-1/2018.

### 2.2 Induction of experimental autoimmune encephalomyelitis

We used the neuroantigen myelin oligodendrocyte glycoprotein 35-55 peptide (MOG35-55) (N-MEVGWYRSPFSRVVHLYRNGK-C) (Genemed Synthesis, Tx, USA) for immunization. The mice were immunized blindly with 100 µg MOG35-55 diluted in 50 µL of phosphate-buffered saline (PBS) emulsified in equal volume with Complete Freund’s Adjuvant (CFA) (Sigma-Aldrich, F5881, MO, USA), which was supplemented with 4 mg/mL of inactivated Mycobacterium tuberculosis (MT, H37RA) (BD Difco, DF3114-33-8, USA). The emulsion was administered to the axillary region of each mouse subcutaneously. Immediately after immunization, and two days later, 200 ng of Pertussis toxin (Sigma-Aldrich, P7208, MO, USA) were injected intraperitoneally per animal. We monitored the clinical signs of EAE daily after the seventh day of immunization. The evolution of the disease was graded blindly as follows: score 0 = not sick; score 1 = loss of tail tone; score 2 = loss of movement of both hind legs, paraplegia; score 3 = paraplegia, loss of abdominal muscles, abdominal weight loss; score 4 = quadriplegia; score 5 = death. Mice at score 4 were closely monitored and a humane endpoint was applied before score 5 was reached. EAE onset was the first day a mouse showed clinical symptoms. Cumulative score corresponded to the sum of scores starting from onset until the last day of observation divided by the number of mice in the group.

### 2.3 Treatment with dimethyl fumarate

We grounded DMF crystals (Sigma-Aldrich, 242926, MO, USA) and mixed them in PBS prepared with sterile tap water to obtain a dose of 7.5 mg/kg. We considered the standard mass of the mice as 20 g. We prepared fresh doses every day and mixed them vigorously before each administration. Mice with EAE were divided into groups receiving 100 µL of the vehicle (PBS) or DMF every 12 h, by gavage, starting from the third day post immunization (d.p.i.). For EAE monitoring, mice were treated daily until the 20^th^ d.p.i. In other experiments, mice were treated daily for either 5, 10, or 15 days, corresponding to the days 7, 12, and 17 d.p.i., respectively. Tissues were collected in the morning after the final gavage for each time point.

### 2.4 Histology

For spinal cord histology on the 21^st^ d.p.i., animals were anesthetized and euthanized, and the spinal cord was removed and immersed in 4% PFA for 48h. The tissues were transferred to a solution of 70% ethanol, and then they were sent to paraffin embedding, and hematoxylin and eosin (HE) staining at Histocell, SP, BR. Additionally, the longitudinal sections of the spinal cord (4 µm thick) were stained with Luxol fast blue-Cresyl violet to visualize myelin. Cellular infiltrates in the HE-stained samples were quantified using ImageJ 2.14.0. The white matter area was delineated, and infiltrates within were identified. Five sections were analyzed for each mouse.

### 2.5 Mesenteric lymph nodes cells

We removed aseptically all mesenteric lymph nodes (mLN), small intestine and colonic mLN, and grounded them against a 70-µm cell strainer to obtain a cell suspension rich in leukocytes.

### 2.6 Quantitative real-time PCR

To obtain the total RNA from mLN cells, we used the Trizol (Invitrogen, 15596018, USA) extraction method, according to the manufacturer’s instructions. The purity and concentration of the nucleic acids were analyzed using nanodrop equipment (Thermo Scientific 2000). We converted the extracted mRNA to cDNA using the High-Capacity cDNA conversion kit (Applied Biosystems, 4368814, USA), also according to the manufacturer’s instructions. For each reaction, an amount of 1 µg of total RNA was added. The cDNA conversion was performed in a thermocycler (Eppendorf AG 6321 BK016651) with the following incubation steps: 25°C for 10 min, 37°C for 2 h, 85°C for 5 min, ending at 4°C.

For analyzing most cytokines and transcription factor expression, we applied the Taqman technique with the LuminoCt^®^ qPCR ReadyMix™ (Sigma-Aldrich, L6669, USA). Briefly, we mixed the diluted (1:20) cDNA with the manufacturer’s master mix in duplicate. We added the following primers for the transcript’s amplification (all from Applied Biosystems, USA): Foxp3 Mm00475162_m1, Tgfb1 Mm01178820_m1, Tnf Mm00443258_m1, Il10 Mm01288386_m1, Ifng Mm01168134_m1, Il-12a Mm01208555_m1, Il17a Mm00439618_m1, and Il27 Mm00461164_m1. As a reference, we used Beta-2-Microglobulin (B2m) Mm00437762_m1. For the expression of IL-6, we used the SyBR Green chemistry with the LuminoCt^®^ SYBR^®^ Green qPCR ReadyMix™ (Sigma-Aldrich, L6544, USA). Primer sequences were Il6 (Forward CACGGCCTTCCCTACTTCAC; Reverse TGCAAGTGCATCATCGTTGT) and B2m (Forward ACAGTTCCACCCGCCTCACATT; Reverse TAGAAAGACCAGTCCTTG CTGAAG), (Exxtend, BR). The expression level was calculated as 2-ΔCt and the data were normalized by the mean expression of the control group.

### 2.7 Flow cytometry

To analyze cytokine production, the mLN cells were incubated with 25 ng/mL Phorbol 12-myristate 13-acetate (PMA), 1 µg/mL Ionomycin, and 5 µg/mL Brefeldin A in RPMI 1640 medium supplemented with 5% of Fetal Bovine Serum (FBS) during 4 h, at 37°C. Then, the cells were stained with extracellular markers, for 30 min, in PBS. For intracellular staining, a permeabilization and fixation buffer (Invitrogen, 00-5523-00, USA) was used. We incubated the cells with the intracellular markers in the permeabilization buffer overnight. The samples were washed, and the data were acquired and analyzed in a BD FACSymphony A5 flow cytometer (BD Bioscience, USA). We analyzed the cytometry data in the FlowJo software. Extracellular antibodies: CD3 PE (555275, BD), CD4 PerCP Cy 5.5 (116012, Biolegend), CD8a PE Cy 7 (100722, Biolegend), CD11c APC Cy 7 (561241, BD), CD11b BV421 (560625, BD), XCR1 APC (148206, Biolegend), CD103 PerCP Cy 5.5 (563637BD). Intracellular antibodies: Foxp3 APC (17577382, eBioscience), IL-10 BV650 (564083, BD), TGFbeta 1,2,3 Alexa 700 (IC1835N, R&D Systems).

### 2.8 Prostaglandin E2 ELISA and cytometric bead assay

The mLN cells (1 x 10^6^ cells/mL) were cultured in RPMI 1640 medium supplemented with 10% FBS and stimulated with lipopolysaccharide (LPS), 0.1 µg/mL, for 24h. The levels of PGE2 in the cell culture supernatant were quantified by ELISA assay (R&D Systems, SKGE004B, USA) according to manufacturer instructions. We quantified the T cells’ cytokines of the undiluted cell culture supernatants using the BD™ CBA Mouse Th1/Th2/Th17 Cytokine Kit (BD, 560485, USA). The acquisition was performed in a BD FACSCalibur flow cytometer (BD Bioscience, USA).

### 2.9 Statistical analysis and sample size calculation

Statistical analyses were performed using non-parametric tests in the GraphPad Prism 9 software. Outliers detected by the Grubbs method (alfa = 0.05) were excluded. The Kruskal-Wallis test was applied to compare three or more groups, and the Mann-Whitney test was used to compare pairs. Differences were considered significant with a P-value < 0.05. The n was determined according to the formula n= 1 + [2C*(s/d)2], for which we considered P < 0.05 (90% power and significance level 0.05 – C value is 10.51). Considering the maximum deviation of 20% and the difference between groups of 50%.

## 3 Results

### 3.1 DMF reduces the severity of experimental autoimmune encephalomyelitis

**Figure 1:**
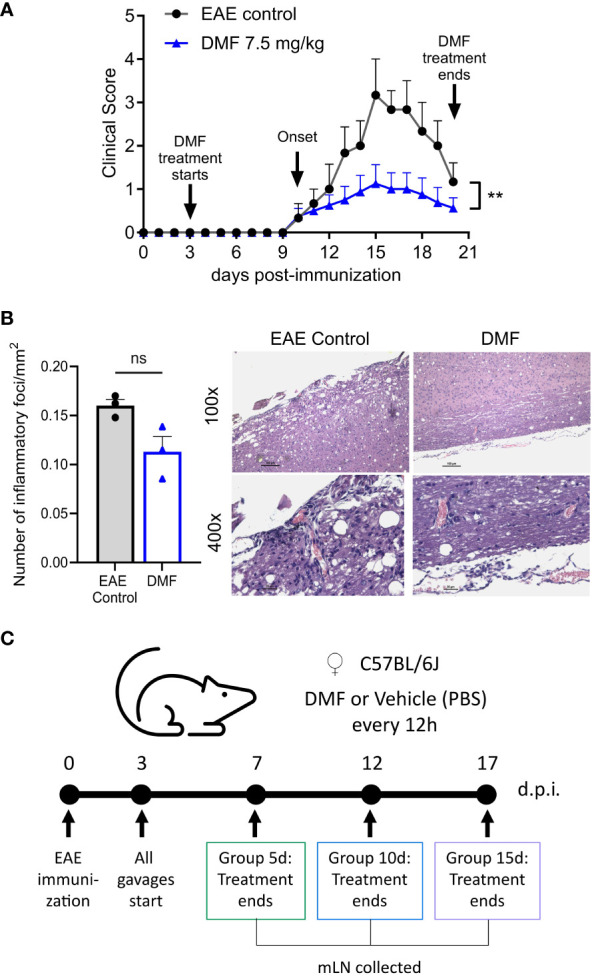
Clinical evolution of EAE in C57BL/6J mice treated with dimethyl fumarate. **(A)** Evolution of EAE after DMF treatment. Representative results out of three experiments are shown. EAE control: without treatment (n = 3); DMF: dimethyl fumarate at a dose of 7.5 mg/kg (n = 8). **(B)** Longitudinal sections of the spinal cord of treated (n=3) and untreated EAE mice (n=3) were stained with HE and the number of inflammatory foci/mm^2^ of white matter in the tissue was counted. Samples were collected 21 days after the immunization. **(A, B)** Data represented as mean and SEM of each group. The Mann-Whitney test was applied for comparison between the EAE control group and DMF treated group. P < 0.05 was considered significant. ** P < 0.01. **(C)** Experimental design for the analysis of mesenteric lymph nodes cells (mLN). Group 5d: mice treated daily for 5 days; Group 10d: mice treated daily for 10 days; Group 15d: mice treated daily for 15 days. All mLN were collected the day after the treatment concluded. D.p.i: days post-immunization, ns: nonsignificant.

Dimethyl fumarate was administrated orally every 12h at 7.5 mg/kg, and PBS was used as a vehicle, following the literature (1, 13–15). **Figure 1A** shows the evolution of clinical signs of EAE for 20 days. The mean onsets of EAE were similar in both the Control (11 ± 1.5) and DMF (11 ± 1.7) groups. The control group had 100% incidence of symptoms, with a maximum score of 3.3 ± 1.2 and a cumulative score of 20.1 per mouse, whereas the DMF group showed a 62.5% of EAE incidence, with a maximum score of 1.1 ± 1.2 and a cumulative score of 8.4 per mouse. These results demonstrate a significant reduction in the severity of EAE in mice receiving 7.5 mg/kg DMF. Additionally, we compared cellular infiltration in the spinal cords of DMF-treated mice with EAE symptoms to those in the EAE control group, observing a trend towards decreased inflammatory infiltration in DMF-treated mice (**Figure 1B**). It should be noted, however, that only the most severely affected mice were selected for HE staining, which could introduce bias to our results by potentially masking a significant reduction in CNS inflammation. A previous study has reported decreased inflammatory infiltration and reduced demyelination with DMF treatment (14). After confirming the protective effects in EAE, we subsequently treated EAE mice with 7.5 mg/kg DMF or the vehicle for 5, 10, or 15 days and analyzed the immune response in the mesenteric lymph nodes (mLN) (**Figure 1C**).

### 3.2 Production of PGE2 by mesenteric cells of C57BL/6J mice with EAE treated with DMF

Flushing and intestinal discomfort are important side effects of DMF treatment. Previous studies demonstrated that the binding of MMF to HCAR2/GPR109A on Langerhans cells promotes the production of Prostaglandin E2 (PGE2), which may explain, at least in part, the flushing effects in the skin (16). The production of PGE2 by macrophages/dendritic cells plays an important role in the pathogenesis of EAE, mainly in the regulation of CD4 Th17 cells (17, 18).

**Figure 2:**
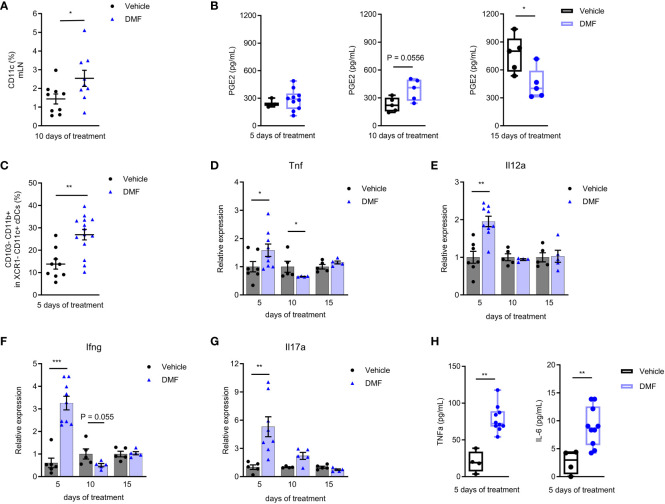
Pro-inflammatory mechanisms are activated in the mesenteric lymph nodes of C57BL/6J mice with EAE at the beginning of treatment with dimethyl fumarate. **(A)** The mesenteric lymph node (mLN) dendritic cells (CD11c+) were stained for flow cytometry. Conventional dendritic cells (cDCs) CD11c+ XCR1- were analyzed for the expression of CD103 and CD11b. **(B, H)** The mLN cells from EAE mice treated or not with 7.5 mg/kg DMF were cultured in RPMI 1640 medium supplemented with 10% FBS and stimulated with lipopolysaccharide (LPS), 0.1 µg/mL, for 24h. **(B)** PGE2 was measured in cell culture supernatant by an ELISA assay. **(C)** CD103-CD11b+ cDCs. **(D–G)** The expression of pro-inflammatory cytokines was measured in the freshly isolated mLN cells by qPCR, having B2m as the housekeeping gene. **(D)** Tnf mRNA. **(E)** Il12a mRNA. **(F)** Ifng mRNA. **(G)** Il17a mRNA. **(H)** The pro-inflammatory cytokines IL-6 and TNF were measured in the cell culture supernatants by flow cytometry using a cytometric bead array kit. n=5-10 for both groups, cohorts are littermates. *P < 0.05, **P < 0.01, ***P < 0.001.

Here, we show an increase in the CD11c+ cells from the mLN (**Figure 2A**) and in the release of PGE2 by mLN cells on the 10^th^ day of treatment with DMF (**Figure 2B**). As the treatment and the disease progress (15^th^), the production of PGE2 in treated mice keeps steady, while there is a significant increase in PGE2 production by mLN cells of EAE mice without treatment. It should be noted that the 17^th^ day of post-immunization (here the 15^th^ day of treatment) usually corresponds to the peak of EAE, when autoreactive T lymphocytes are circulating throughout the body of mice, leading to systemic inflammation (19).

### 3.3 Initial immune response in the mLN of C57BL/6J mice with EAE treated with DMF

EAE had long been considered the prototypic of both Th1 and Th17-mediated autoimmune disease (20). Here, we demonstrate that the initial treatment with DMF stimulates a pro-inflammatory environment in the gut. We analyzed the conventional DCs type 2 from the mLN, which are CD11c+ XCR1- (21), by flow cytometry. We observed an increase in the CD103- CD11b+ DC population (**Figure 2C**) after 5 days of treatment. Intestinal CD103- DCs might migrate to mLN and promote the production of pro-inflammatory cytokines by effector cells (22).

The treatment with DMF initially favored the expression of innate immune response pro-inflammatory cytokines in the mLN, such as TNF (**Figure 2D**) and IL-12a (**Figure 2E**) mRNA. In addition, we saw an expressive increase in the production of IFN-γ (**Figure 2F**) and IL-17A (**Figure 2G**) mRNA within 5 days of treatment. The expression of IL-6 mRNA was similar to the control group during the analysis period (**Supplementary Figure 4A**).

The mLN cells were also stimulated *ex-vivo* with LPS for 24h to evaluate cytokine production. The levels of TNF and IL-6 (**Figure 2H**) were higher in cells from DMF-treated animals after 5 days of treatment. There were no changes in other Th1, Th2, and Th17 cytokines levels after the LPS stimulus (**Supplementary Figure 4B–F**).

**Figure 3:**
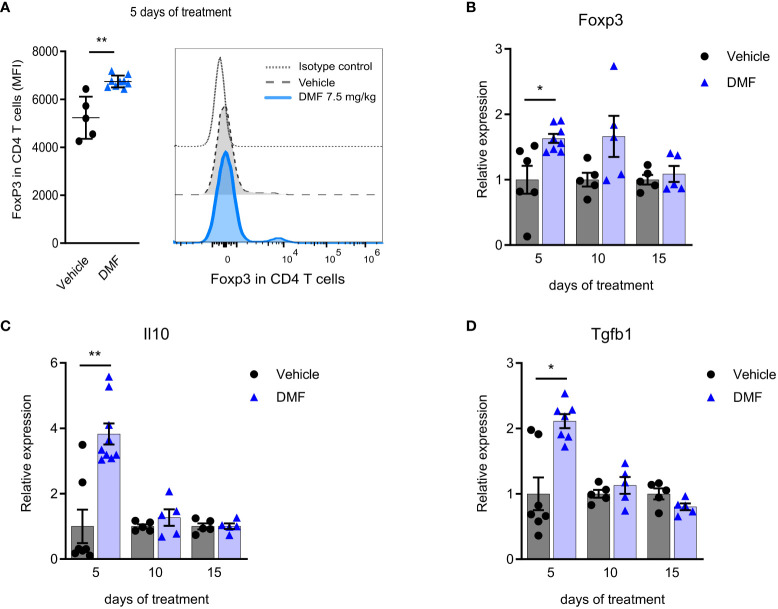
An anti-inflammatory response in the mesenteric lymph nodes of C57BL/6J mice with EAE accompanies the pro-inflammatory stimuli in the initial phase of dimethyl fumarate treatment. CD4 T lymphocytes from freshly isolated mesenteric lymph nodes (mLN) were analyzed by flow cytometry. **(A)** Median fluorescence intensity of Foxp3 in CD4 T cells. The expression of T reg-associated molecules was measured in the directly processed mLN cells by qPCR, having B2m as the housekeeping gene. **(B)** Foxp3 mRNA; **(C)** Il10 mRNA; **(D)** Tgfb1 mRNA; *P < 0.05; n=5-10 for both groups, cohorts are littermates. **P < 0.01.

Interestingly, this initial pro-inflammatory profile is accompanied by regulation in DMF-treated mice. The oral administration of DMF increases the Foxp3 expression on CD4 T lymphocytes in mLN by the 5^th^ day (**Figure 3A**). In addition, higher transcription of Foxp3 (**Figure 3B**), IL-10 (**Figure 3C**), and TGF-β (**Figure 3D**) by mLN cells are observed. A small population of Foxp3 CD4 T cells producing TGF-β increased during 5 days of DMF treatment (data not shown). We attribute this result to the characteristics of DMF. Within the intestine, DMF is transformed into its active metabolite MMF, which binds to the HCAR2 receptor on dendritic cells. It is well described in the literature that small-chain fatty acids, such as butyrate, utilize this same HCAR2 receptor to reduce the inflammatory response in the intestine and promote the activation of the CD4+Foxp3+ Treg population (23, 24). Moreover, Round and Mazmanian have demonstrated that oral administration of a fraction of *Bacteroides fragilis* leads to an increase in CD4+Foxp3+ T reg in mesenteric lymph nodes in mice, and either CD4+Foxp3+ or CD25+Foxp3+ had a regulatory function (25).

After a few days, the expression of pro- and anti-inflammatory molecules was similar in DMF and control groups. In fact, by the 10^th^ day, TNF transcription even decreased in DMF-treated mice (**Figure 2D**), and IFN-γ expression was also reduced (**Figure 2F**).

### 3.4 Late immune response in the mLN of C57BL/6J mice with EAE treated with DMF

**Figure 4:**
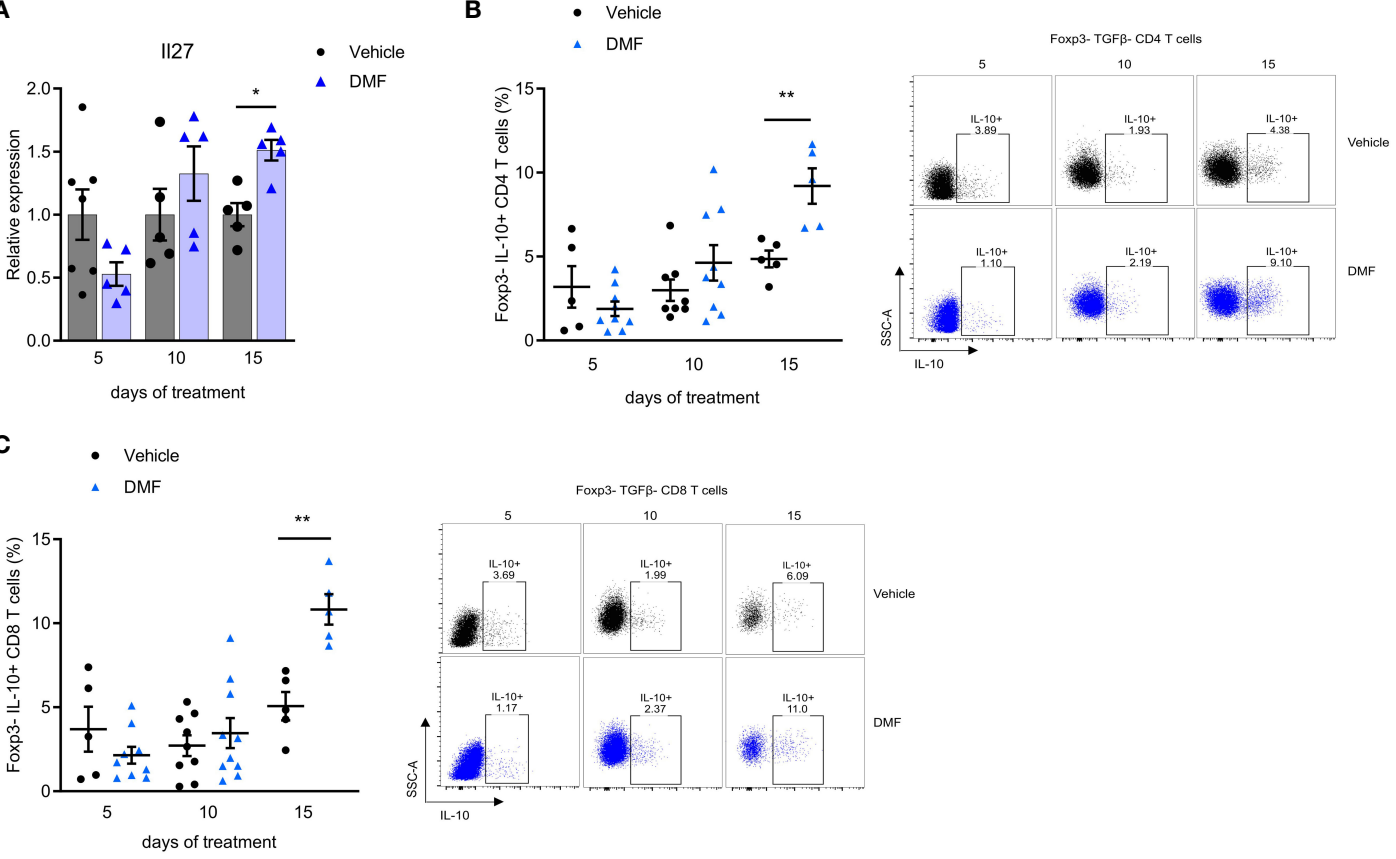
An anti-inflammatory response predominates in the mesenteric lymph nodes of C57BL/6J mice with EAE as the treatment with dimethyl fumarate progresses. Mesenteric lymph nodes (mLN) cells from immunized mice were directly prepared for total RNA extraction and the qPCR protocol. **(A)** Il27 mRNA expression in the mesenteric lymph nodes (mLN) by qPCR, having B2m as the housekeeping gene. For analyzing the expression of IL-10 in T cells, a fraction of mLN cells were stimulated ex-vivo with PMA, Ionomycin, and Brefeldin A for 4h. T lymphocytes from the mLN were analyzed by flow cytometry. **(B)** Regulatory T cells in CD4+ Foxp3- population expressing IL-10 (Tr1). **(C)** Regulatory T cells CD8+ Foxp3- population expressing IL-10. n=5-10 for both groups, cohorts are littermates. *P < 0.05, **P < 0.01.

Further during treatment, the DMF stimulated an anti-inflammatory environment in the mLN of C57BL/6J mice. We observed an increase in the expression of IL-27 (after 15 days). IL-27 is a pleiotropic cytokine released by antigen-presenting cells (APC), which modulates several immune cells, including myeloid cells and T cells. A significantly higher IL-27 expression was observed in the group that received DMF for 15 days (**Figure 4A**).

Our findings demonstrate that oral administration of DMF induces an increase of regulatory T cells of gut-draining lymph nodes of C57BL/6J mice with EAE. A significant increase in IL-10-producing CD4 T lymphocytes was observed after 15 days of treatment (**Figure 4B**). There is no expression of Foxp3 in this cell population, suggesting that it belongs to the Tr1 subset of regulatory T cells (26). We also showed a significant increase in IL-10 production by Foxp3^-^ CD8 T lymphocytes after 15 days of treatment (**Figure 4C**). The proportion of Foxp3 T reg cells was the same in treated and control mice during the observed period (data not shown).

## 4 Discussion

Here we demonstrate that DMF reduces the severity of EAE and gradually induces regulatory T cells in the mesenteric lymph nodes. Our data agree with previous observations that the treatment with DMF significantly reduces the severity of EAE (13, 14). The reduction of the severity of EAE may be explained, at least in part, by the ability of monomethyl fumarate (MMF), the bioactive metabolite of DMF, to cross the blood-brain barrier and downregulate the microglia activation (3).

The MMF is a potent agonist for hydroxycarboxylic acid receptor 2 (HCAR2) (4), which may either reduce the inflammatory response (3) or increase the inflammation in both the skin and gastrointestinal tract (8). The pro-inflammatory effect of MMF can be related to the activation of the COX-2 pathway in skin flushing under DMF administration (16, 27). Prostaglandins (PGs) are important in maintaining gastrointestinal homeostasis by contributing to the maintenance of mucosal integrity and limiting the inflammatory response (28). However, increased PG production occurs within the gastrointestinal mucosa of patients with inflammatory bowel disease (IBD) and in experimental models of IBD (29, 30). Treatment with DMF in a mouse model of colitis showed reduced COX-2 levels in the colon of treated mice, which were associated with the alleviation of colitis (31). Moreover, previous studies have demonstrated profound alterations in PGE2 levels in CNS-derived samples from animals with EAE (17, 18) indicating that PGE2 plays a role in the pathogenesis of the disease. In agreement with these observations, we demonstrated a significant increase of PGE2 by DC cells from mesenteric lymph nodes of mice at the peak of EAE when the inflammatory response is systemic, while the PGE2 levels of DMF-treated mice increase at the beginning of the treatment and later remain significantly reduced compared to the control animals, suggesting both pro-inflammatory and anti-inflammatory effects of DMF in the gut draining lymph nodes.

Besides the modulation of PGE2 in the mesenteric lymph nodes, we observed the increase of innate immune response cytokines, such as TNF, IL-12, and IL-6, and pro-inflammatory cytokines from lymphoid cells, such as IFN-g and IL-17A, in mice treated for five days with DMF. It has been shown that gut CD103- CD11b+ DCs can activate T cells and induce the expression of Th1 and Th17-related cytokines (22). We observe an increase in the population of those DCs after five days of treatment, which might induce the higher expression of IFN-γ and IL-17A mRNA. The expression of TGF-β and IL-10 was also higher in mice treated for five days with DMF, reinforcing that DMF treatment induces either pro-inflammatory cytokines or anti-inflammatory response in the initial phase of treatment.

In acute colitis models, DMF appears to promote a reduction in inflammatory activity within just 7 to 10 days of treatment. Previous studies have showed reductions in NF-κB, IL-6, IL-17a, and TNF levels (32, 33), as well as decreased oxidant activity (34) in the colonic lamina propria of DMF treated mice. It is noteworthy that these models induce severe damage to the epithelial barrier, leading to increased interaction between microbiota and lamina propria immune cells, thereby triggering an inflammatory response. In EAE, barrier dysfunction has also been reported, particularly in the early stages of the disease, although it is not as severe as in colitis models (35, 36). The intensity of stimuli in colitis models could contribute to favoring the activation of antioxidant pathways of DMF, as there is evidence of a dependency on Nrf2 for the alleviation of colitis (31, 34). However, studies with Nrf2 knockout mice in EAE have shown that DMF treatment can decrease disease severity even in the absence of the Nrf2 molecule (37), indicating this may not be a primary mechanism of action for DMF in the EAE model. Thus, the balance towards pro-inflammatory factors that we observed during the early stages of EAE in the gut draining lymph nodes may suggest that DMF could exert different effects in the gut depending on local environmental cues. Future studies with lamina propria immune cells could help to elucidate the specific pathways activated by DMF in the gut during EAE. As the treatment progressed, a significant increase of IL-10- producing type 1 regulatory T cells (Tr1 cells) was observed, as well as IL-27, while pro-inflammatory cytokines maintained the same levels as the control. The initial activation of Foxp3+ CD4 T cells may modulate the posterior generation of Tr1 cells since a previous study demonstrated that Foxp3+ T regs condition DCs to induce IL-10-secreting Tr1 cells by a mechanism dependent on IL-27 (38). IL-27 suppresses Th1 and Th17 responses and limits CNS inflammation in several experimental models (39). Consequently, the *in vivo* administration of IL-27 inhibits EAE development. In addition, the inhibition of IL-27 signaling results in exaggerated Th17 responses and the worsening of EAE (39, 40). IL-27 has been shown to inhibit the development of Th17 cells and to promote the differentiation of Tr1 cells (39, 41). Our data show that the increased production of IL-27 by mesenteric lymph node cells on the fifteenth day of treatment with DMF reduced both IL-17 levels, that were practically normal on the tenth day of treatment, and PGE2.

The IL-27 signaling may also induce the expression of IL-10 on CD8 T lymphocytes (42). Here, we see an increase in IL-10+ CD8 T cells in the gut lymph nodes with the continuation of DMF treatment. The production of IL-10 by CD8 T cells is enhanced in the CNS during the chronic phase of EAE (19), which might contribute to the control of the inflammatory immune response associated with the disease. In addition, a subset of CD122 CD8 regulatory T cells can express high amounts of IL-10 when stimulated by activated T cells and appear to regulate effector lymphocytes in the lymphoid organs (43, 44). These CD122 CD8 T regs reduce the severity of EAE after adoptive transference (45). Therefore, the cells we observed probably contribute to the anti-inflammatory microenvironment in the gut and may participate in the protection of EAE.

In the past decades, studies demonstrated that T cells in patients with autoimmune diseases such as MS might become refractory to T reg suppression (46). Many resistance-inducing mechanisms of effector T cells to suppress T regs have been associated with increased proinflammatory cytokines such as IL-6 and TNF (47, 48). However, another study reveals that DMF therapy enhances the responsiveness of the T effector to T reg in MS patients (49). In line with these observations, our data demonstrate that treatment of mice with EAE with DMF significantly reduces the expression of TNF and does not modify IL-6 levels in the gut draining lymph nodes in the 15^th^ day of treatment, which may contribute to the responsiveness of the T effector to T regs generated in the intestinal mucosa.

Like all studies with disease models, we must acknowledge the primary limitations of our work. Although it appears that DMF treatment operates through various mechanisms over time in MS patients, directly correlating the duration of DMF treatment in humans with the treatment period in EAE is not feasible. One reason for this is the markedly different disease courses. Here, we consider the initial days of DMF treatment primarily as a five-day continuous treatment, aligning with the early stages of EAE (up to the 7th d.p.i), before disease onset. In MS patients the disease progresses over years, so the initial period could be considered in terms of months. Another challenge we encountered was determining a DMF dose that could be comparable to the dosage used by MS patients. Typically, an adult takes 240 mg of DMF every 12h. Initially, we tried different concentrations of DMF, ranging from 7.5 to 150 mg/kg. However, at the higher concentrations, we observed intestinal discomfort (diarrhea) in the animals. Nonetheless, these doses were considerably higher compared to those administered to MS patients. We therefore reduced the DMF dose as much as possible (7.5 mg/kg), which is still high. At this dosage, we observed a decrease in disease severity without the discomfort associated with higher doses. Thus, while we observed early pro-inflammatory activity induced by DMF, we were unable to directly correlate it with gastrointestinal symptoms in the mice.

Taken together, this study demonstrated that the administration of DMF to animals with EAE increases the inflammatory response with an increase in the population of CD103- dendritic cells and the production of pro-inflammatory molecules. After five days of treatment, there is also an increase in CD4+Foxp3+ lymphocytes and anti-inflammatory cytokines, which is followed by a consistent increase in IL-27 and Tr1 regulatory cells. These mechanisms may explain the adverse effect of DMF treatment and its resolution. Our data suggest that the intestinal mucosa may be the first microenvironment of generation of regulatory T cells in DMF-treated MS patients, which may contribute to reducing the inflammatory response.

## Data availability statement

The raw data supporting the conclusions of this article will be made available by the authors, without undue reservation.

## Ethics statement

The animal study was approved by UNICAMP Animal Ethics Committee (CEUA/UNICAMP), 4884-1/2018, Universidade Estadual de Campinas, Campinas, Brazil. The study was conducted in accordance with the local legislation and institutional requirements.

## Author contributions

AL: Conceptualization, Data curation, Formal Analysis, Investigation, Methodology, Project administration, Validation, Visualization, Writing – original draft, Writing – review & editing. BF: Data curation, Formal Analysis, Investigation, Methodology, Project administration, Writing – review & editing. FP: Data curation, Formal Analysis, Investigation, Methodology, Validation, Writing – original draft, Writing – review & editing. RC: Methodology, Validation, Writing – review & editing. SR: Methodology, Validation, Writing – review & editing. RQ: Methodology, Writing – review & editing. MS: Methodology, Writing – review & editing. NB: Methodology, Writing – review & editing. AM: Methodology, Writing – review & editing. IS: Methodology, Writing – review & editing. AF: Formal Analysis, Supervision, Writing – review & editing. EO: Formal Analysis, Investigation, Methodology, Supervision, Validation, Writing – review & editing. LS: Formal Analysis, Funding acquisition, Investigation, Project administration, Resources, Supervision, Visualization, Conceptualization, Writing – original draft, Writing – review & editing.
