# Supplementary material for: Dimethyl fumarate modulates the regulatory T cell response in the mesenteric lymph nodes of mice with experimental autoimmune encephalomyelitis
Source: Front Immunol. 2024 May 3;15:1391949. doi: 10.3389/fimmu.2024.1391949 (PMC11099268; doi:10.3389/fimmu.2024.1391949)
Supplement: Supplementary file 1 [file DataSheet_1.pdf]

Supplementary Material

Dimethyl Fumarate modulates the regulatory T cell response in the Mesenteric Lymph Nodes of Mice with Experimental Autoimmune Encephalomyelitis

Supplementary figures

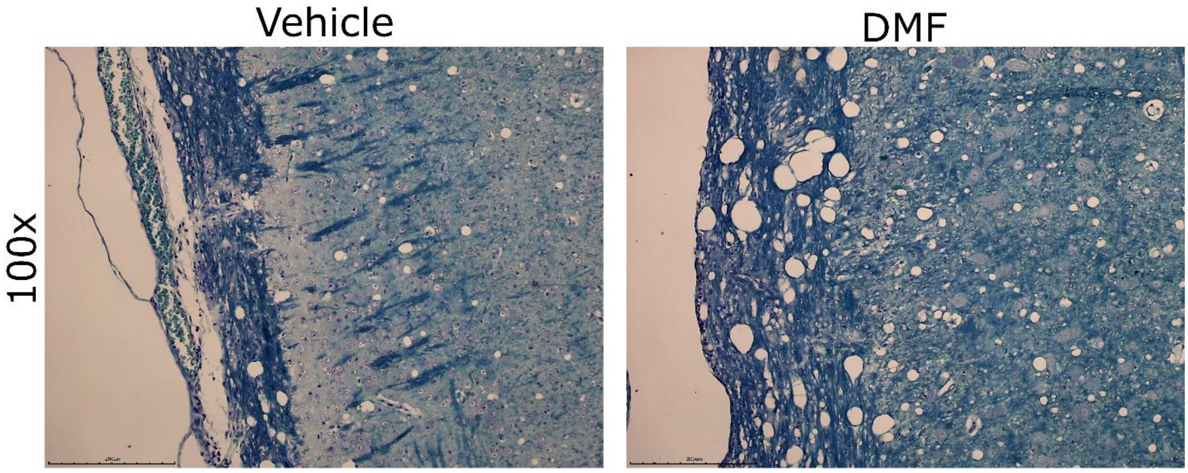

Supplementary Figure 1. Luxol Fast Blue-Cresyl Violet Staining of spinal cord from EAE mice.

Mice which developed EAE symptoms had their spinal cord collected and preserved for paraffin-embedding at day 21st post-immunization (Control (Vehicle), n=3; DMF, n=3). Longitudinal sections (4 µm) were stained with Luxol Fast Blue-Cresyl Violet to observe myelin content. Representative images are shown.

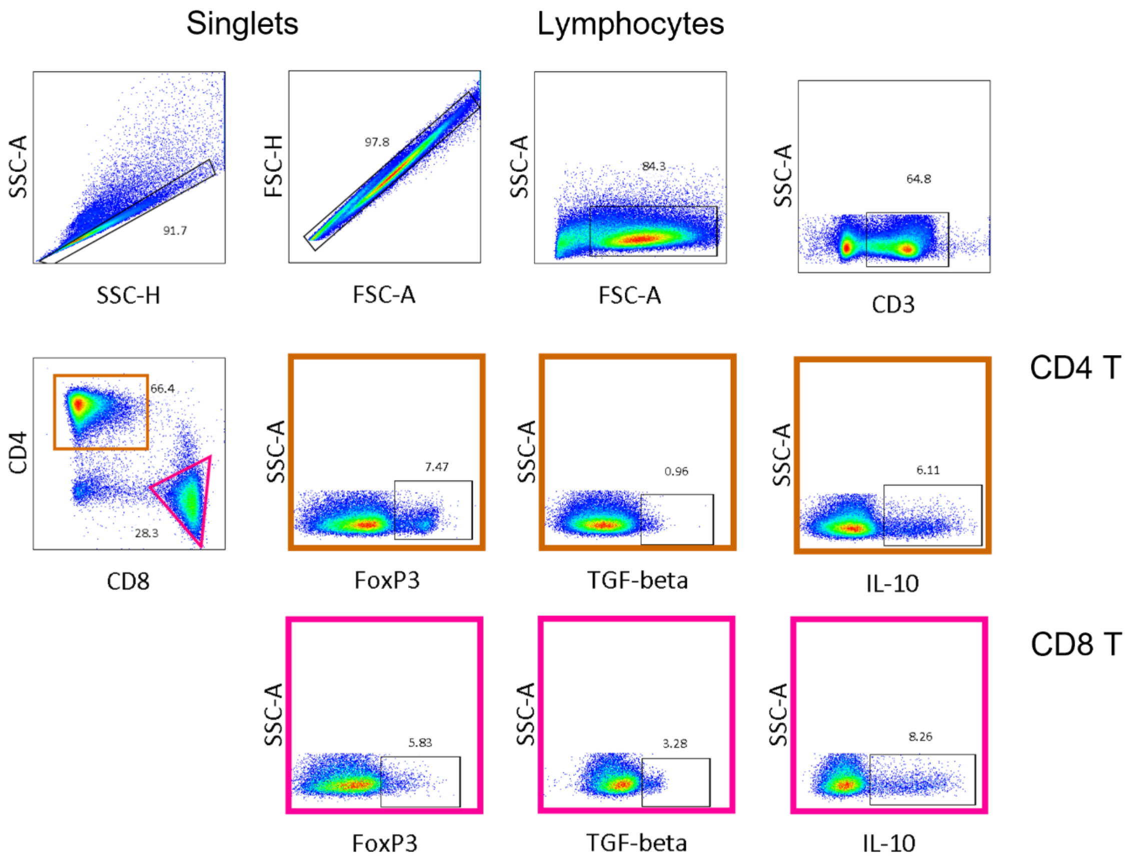

Supplementary Figure 2. Flow cytometry gate strategy for analyzing regulatory T cells.

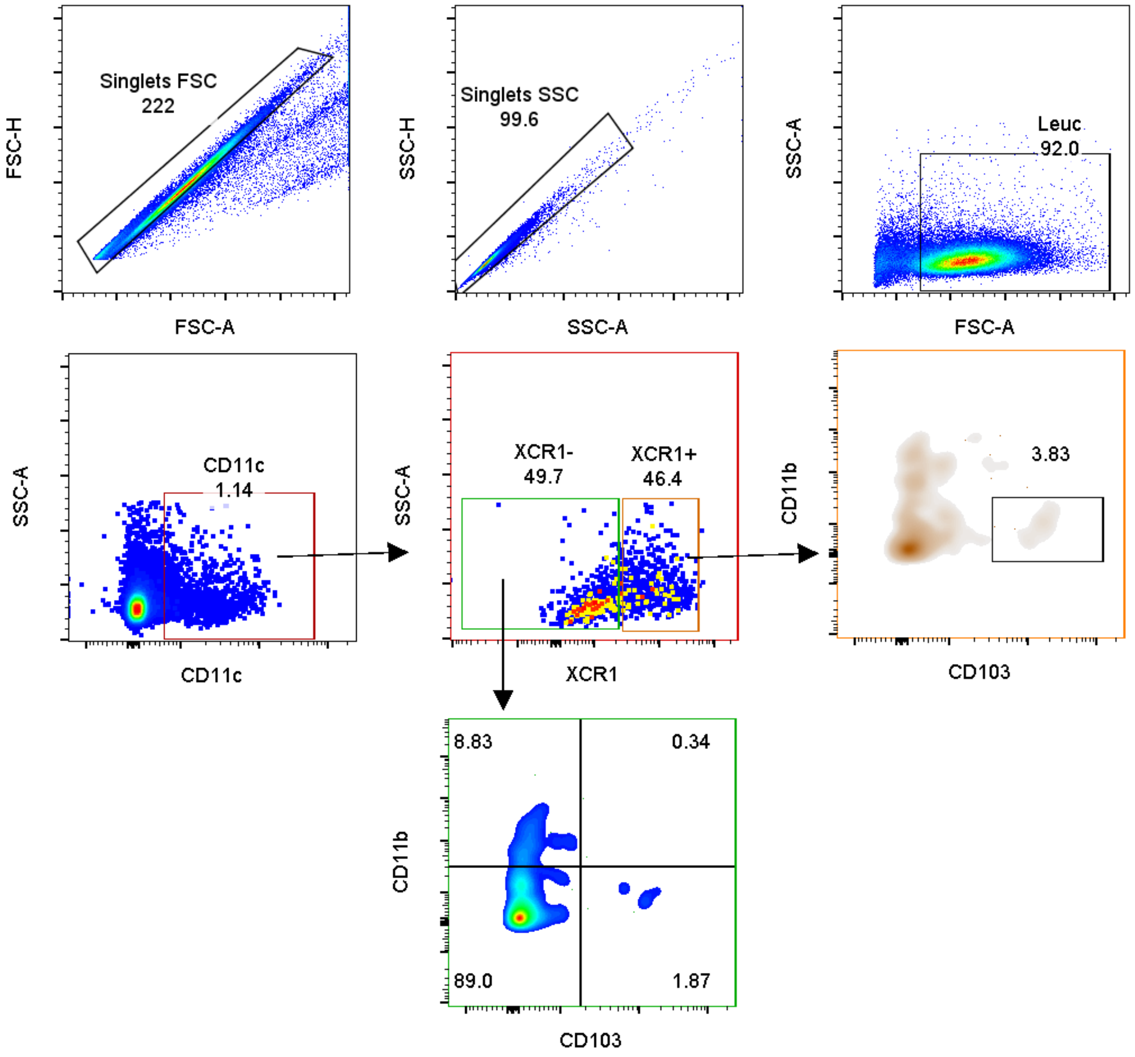

Supplementary Figure 3. Flow cytometry gate strategy for analyzing dendritic cells.

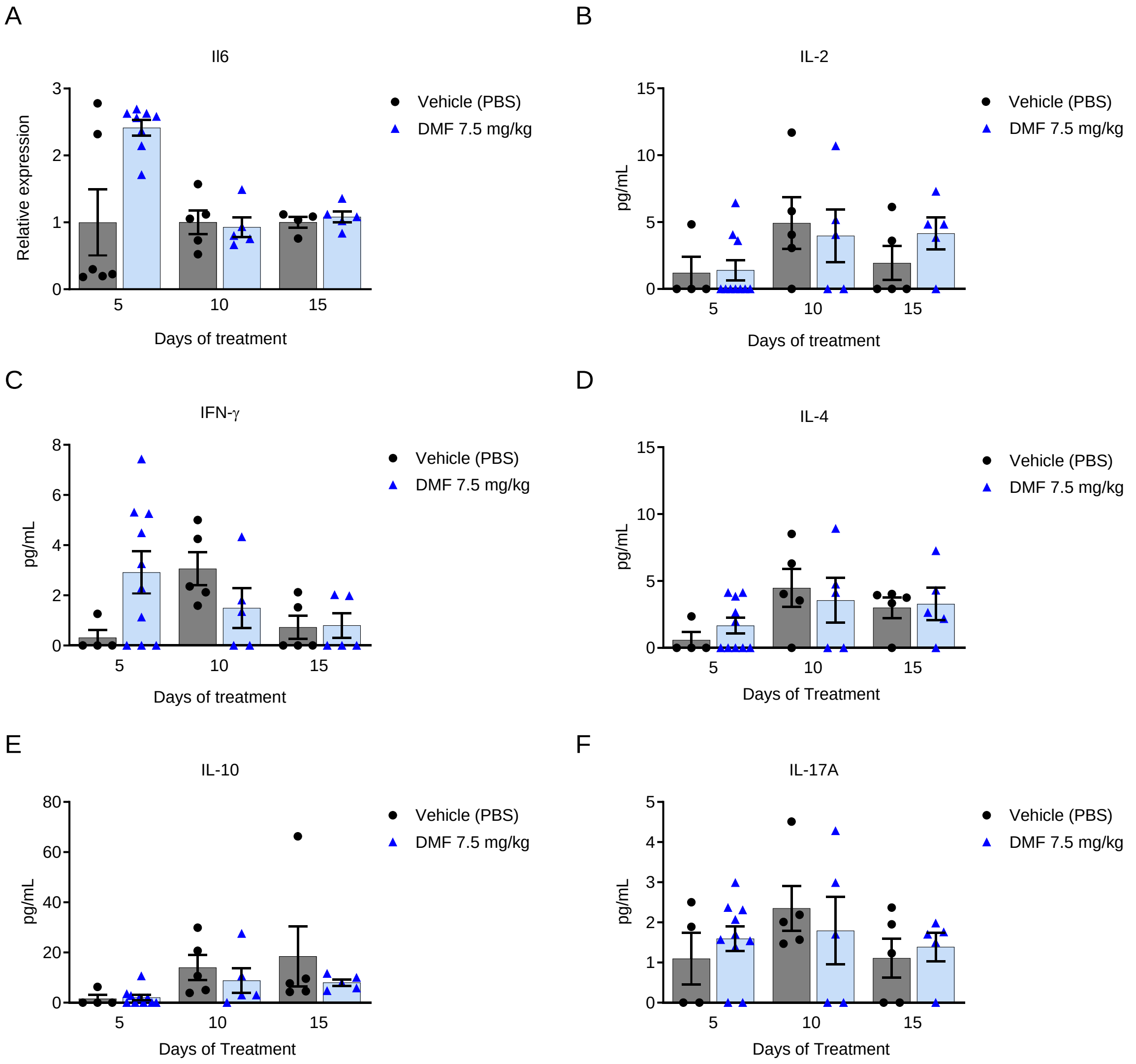

Supplementary Figure 4. Cytokines produced by mesenteric lymph nodes cells (mLN) during treatment with dimethyl fumarate of mice with EAE.

The expression of pro-inflammatory cytokines was measured in the fresh mLN cells by qPCR, having B2m as the housekeeping gene. (A) Il6 mRNA. The mLN cells of mice with EAE receiving DMF 7.5 mg/kg or vehicle were cultured in RPMI 1640 medium supplemented with 10% FBS and stimulated with lipopolysaccharide (LPS), 0.1 µg/mL, for 24h. Pro-inflammatory cytokines were measured in the supernatant by flow cytometry using a cytometric bead array kit. Levels of (B) IL-2, (C) IFN-γ, (D) IL-4, (E) IL-10, and (F) IL-17A.
